# Role of MicroRNAs 99b, 181a, and 181b in the Differentiation of Human Embryonic Stem Cells to Vascular Endothelial Cells

**DOI:** 10.1002/stem.1026

**Published:** 2012-01-09

**Authors:** Nicole M Kane, Lynsey Howard, Betty Descamps, Marco Meloni, John McClure, Ruifang Lu, Angela McCahill, Christopher Breen, Ruth M Mackenzie, Christian Delles, Joanne C Mountford, Graeme Milligan, Costanza Emanueli, Andrew H Baker

**Affiliations:** a British Heart Foundation Glasgow Cardiovascular Research Centre, Institute of Cardiovascular and Medical Sciences, University of GlasgowGlasgow, United Kingdom; b Experimental Cardiovascular Medicine Division, Bristol Heart Institute, University of BristolBristol, United Kingdom; c Institute of Neuroscience and Psychology, College of Medical, Veterinary and Life Sciences, University of GlasgowGlasgow, United Kingdom

**Keywords:** MicroRNA, Vascular endothelial cells, Embryonic stem cells, Differentiation, Regenerative medicine

## Abstract

MicroRNAs (miRNAs) are short noncoding RNAs, which post-transcriptionally regulate gene expression. miRNAs are transcribed as precursors and matured to active forms by a series of enzymes, including Dicer. miRNAs are important in governing cell differentiation, development, and disease. We have recently developed a feeder- and serum-free protocol for direct derivation of endothelial cells (ECs) from human embryonic stem cells (hESCs) and provided evidence of increases in angiogenesis-associated miRNAs (miR-126 and -210) during the process. However, the functional role of miRNAs in hESC differentiation to vascular EC remains to be fully interrogated. Here, we show that the reduction of miRNA maturation induced by Dicer knockdown suppressed hES-EC differentiation. A miRNA microarray was performed to quantify hES-EC miRNA profiles during defined stages of endothelial differentiation. miR-99b, -181a, and -181b were identified as increasing in a time- and differentiation-dependent manner to peak in mature hESC-ECs and adult ECs. Augmentation of miR-99b, -181a, and -181b levels by lentiviral-mediated transfer potentiated the mRNA and protein expression of EC-specific markers, Pecam1 and VE Cadherin, increased nitric oxide production, and improved hES-EC-induced therapeutic neovascularization in vivo. Conversely, knockdown did not impact endothelial differentiation. Our results suggest that miR-99b, -181a, and -181b comprise a component of an endothelial-miRNA signature and are capable of potentiating EC differentiation from pluripotent hESCs

## INTRODUCTION

MicroRNAs (miRNAs) are highly conserved, short noncoding RNA molecules of approximately 22 nucleotides in length in their mature form. miRNAs originally reside in the nucleus as RNA polymerase II primary miRNA (pri-miRNA) transcripts [1]; thereafter, they are processed in two steps. Within the nucleus, pri-miRNAs are processed into approximately 70–100 nucleotide hairpin-shaped precursors known as pre-miRNAs by Drosha, which form complexes with a double-stranded RNA-binding domain protein, DGCR8/Pasha [2, 3], before being transported to the cytoplasm via an Exportin-5- and Ran-GTP-dependent mechanism [4]. Once in the cytoplasm, the double-stranded ribonuclease, Dicer, cleaves the pre-miRNAs to yield approximately 20–22 base pair mature miRNA duplexes [5]. The duplex is incorporated into the effector ribonucleic acid-induced silencing complex (RISC) where the duplex is unwound to form a mature single-stranded miRNA. The mature miRNA strand that is incorporated in the RISC binds to the 3′ untranslated region and more rarely to other regions of specific target mRNAs through a perfect or near-perfect complementary match to block translation or cleaves the mRNA [6, 7] (reviewed in [8–10]).

Pluripotent stem cells can be directly differentiated toward endothelial cell (EC) lineages [11] and may be used to generate a large supply of transplantable, healthy, functional cells for repair of ischemic tissues. However, the control of the intricate transcriptional events controlling pluripotent stem cell to vascular EC differentiation remains to be fully interrogated. Pluripotent human embryonic stem cells (hESCs) are reported to express a unique panel of miRNAs, including the miR-302 cluster and miR-371/372/373 cluster [12, 13]. The expression of these pluripotency-associated miRNAs is suppressed upon differentiation with concordant induction of differentiation-specific miRNAs, namely miR-145 and Let7 [14, 15]. Indeed, it has recently been demonstrated that overexpression of the miR-302 cluster can facilitate reprogramming of somatic cells to pluripotency [16]. We believe that pluripotent stem cells, both hESCs and induced pluripotent stem cells, may provide a unique model system through which the study of miRNA function during early stages of lineage development and commitment can be quantified.

Initial evidence for contribution of miRNAs in EC development and differentiation was provided by the observation that Dicer knockout mice have an embryonic lethal phenotype [17]. In fact, Dicer knockout embryos suffered severely disrupted blood and vessel formation accompanied with retarded expression of early endothelial markers VEGF, FLT1, KDR, and tie1 [17]. These results were supported in vitro by demonstration that Dicer is a prerequisite for EC function [18, 19]. However, endothelial-restricted tissue-specific deletion of Dicer demonstrated that endothelial progenitor cells developed, albeit with compromised blood circulation, and a lack of embryonic lethal phenotype was observed in both zebrafish [20] and mice [21]. These results suggest that miRNAs may not be pivotal in endothelial differentiation and developmental angiogenesis. This is compounded by the failure to date in using a specific miRNA deletion to evoke fully penetrant embryonic lethality in mice. On other hand, murine ESCs subjected to loss of global miRNAs via Dicer or Drosha knockdown exhibit reduced self-renewal and proliferative capabilities with marked defects in differentiation [22, 23]; however, this has yet to be reported in hESCs. To date, only a small number of studies have focused on the involvement of angiogenesis-associated miRNAs in EC differentiation from pluripotent stem cells. This is in agreement with current literature reporting that despite the demonstration of differentially expressed miRNAs during endothelial differentiation in vitro, none of the miRNAs so far have been reported to directly govern EC fate decisions [24, 25]. For example, miR-126 was identified as enriched in ECs derived from mouse ESCs and in developing mouse embryos [26, 27], with knockdown studies in zebrafish resulting in hemorrhage and loss of vascular integrity [26, 27]. Indeed, previous studies in zebrafish have provided evidence that miR-126 provides a crucial link between flow and Vegf signaling to promote angiogenesis, by acting downstream of klf2a to drive shear flow-stimulated angiogenesis via repression of spred1 and pik3r2 [28]. Furthermore, targeted deletion of miR-126 evoked defects in EC function and resulted in leaky vessels in mice, but did not evoke full embryonic lethality [29], suggesting that the endothelial-specific miR-126 does not control EC differentiation but does control cell function [25]. This hypothesis is further ratified with results from a previous study reporting that miRNA-126 overexpression in pluripotent SCs prevented differentiation toward the EC lineage [27], indicating that miR-126 does not command early EC lineage commitment but is induced by vascular differentiation.

We have recently described the increased expression of angiogenesis-associated miRNAs (miR-126, -130a, -133a, -133b, and -210) [25, 30] and decrease in antiangiogenic miRNAs (miR-20a, -20b, -221, and -222) [31] during our feeder- and serum-free-directed hESC-EC differentiation protocol [11]. To date, despite the demonstration of miRNA regulation in a myriad of vascular biology events and the mapping of initial stages of mesoderm commitment [32], no individual miRNAs have been shown to directly control mesoderm to vascular EC fate decisions. There is still insufficient evidence demonstrating miRNA involvement in early human development of vascular cells, how they control mesoderm-EC fate commitment, and the mechanisms involved in these differentiation processes.

In this study, we analyzed early stages of EC lineage commitment and the miRNA-ome to identify miRNAs integral to human EC development. We identified miR-99b, -181a, and -181b as miRNAs enriched in hES-ECs differentiated from pluripotent stem cells and confirmed their expression in adult venous ECs. We interrogated their role in differentiation by modulating their expression and directing differentiation to EC lineage. Overexpression of the aforementioned miRNAs augmented the differentiation potential of pluripotent cells and improved their potential for therapeutic angiogenesis in a mouse model of limb ischemia. The results herein demonstrate that miRNA regulation of development to specified lineages is a potential target for optimizing the generation of proangiogenic vascular ECs for regenerative medicine.

## MATERIALS AND METHODS

See also Supporting Information Detailed Methods online for further reference.

### Cell Lines and Culture Conditions

The hESC lines H1, H9 (WiCell Research Institute, Madison, WI, http://www.wicell.org), and 461 (Cellartis, Dundee, U.K.) were cultured in a feeder-free system [11]. Endothelial differentiation was induced as previously described [11]. Hematopoietic and neural progenitor cells were generated from H1 hESC as previously described [33, 34]. The cells were harvested at defined time points as indicated and expressed characteristic markers of hESC-derived hematopoiesis (hES-hematopoietic progenitor cell [HPC] CD34 [41%] and CD45 [54%]) as analyzed by flow cytometric analysis. Samples from neuronal differentiations (hES-neural) were taken at day 10 for consistency with other miRNA analyses at this time point. Cultures performed in this lineage differentiation display characteristic early neural rosette morphology and later entangled projections and positive staining for PAX6 and nestin as previously described [34]. Primary human saphenous vein ECs (SVEC) were cultured in endothelial differentiation media supplemented with 20% fetal bovine serum (FCS) (PAA Laboratories, Somerset, U.K., http://www.paa.at). HEK293T cells (ATCC, Teddington, U.K., http://www.atcc.org) were maintained in Dulbecco's modified Eagle's medium (Invitrogen, Paisley, U.K., http://www.invitrogen.com) supplemented with 10% heat-inactivated FCS (Invitrogen), penicillin (50 μg/ml; Invitrogen), streptomycin (50 μg/ml; Invitrogen), l-glutamate (2 mM; Invitrogen), and sodium pyruvate (1 mM; Invitrogen). All cells were cultivated at 37°C in a humidified atmosphere containing 5% CO_2_.

### Lentiviral Vectors and hESC Infection

Lentiviral (LV) vectors were produced as previously described [35]. LV titers were ascertained by TaqMan quantitative real-time polymerase chain reaction (Q-RT PCR) [11, 36]. For infection, 2 × 10^4^ cells were transduced with a multiplicity of infection (MOI) of 25. Cells were incubated in pluripotent maintenance media containing LV particles and 4 μg/ml polybrene (Sigma-Aldrich, Dorset, U.K., http://www.sigmaaldrich.com) for 18 hours at 37°C in a humidified atmosphere containing 5% CO_2_. LV particles were removed, and media were replaced with fresh pluripotent maintenance media for an additional 24 hours to permit cell recovery.

### Overexpression of miRNAs

The pre-miRNA sequence of -99b, -181a, or -181b (Supporting Information Table 1) was cloned directly into the lentiviral construct (pLNT)/spleen focus-forming virus (SFFV)-multiple cloning site (MCS), plasmid (kind gift from Prof. Adrian J. Thrasher, ICH, UCL, London, U.K.) to obtain the construct pLNT/SFFV-premiR. Suppression of miRNA function was achieved by a miRNA Zip plasmid specific for miR-99b, -181a, and -181b (Cambridge Bioscience, Cambridge, U.K., http://www.bioscience.co.uk). Dicer knockdown was achieved with pSicoR human Dicer plasmid (kind gift from Prof. Richard I. Gregory, Harvard, MA) [37].

### miRNA Microarray

The miRNA expression profile of hESC-ECs was determined by two-channel miRNA microarray analysis using the human miRNA array (LC Sciences, Houston, TX) based on miRBase, version 10.1 (Sanger miRBase) (http://www.ncbi.nlm.nih.gov/geo/query/acc.cgi?acc=GSE33675). Supporting Information Figure S1 depicts the experimental design of the miRNA microarray. To assess the statistical significance of intergroup differences, the empirical Bayes method implemented in the limma R package was used [38]. Significance was assessed using the false discovery rate multiple rate multiple testing correction method, with a false discovery rate cutoff of 5%.

### TaqMan Q-RT PCR Analysis of Mature miRNAs and mRNAs

Total cellular RNA was isolated using miRNAeasy Mini Kit (Qiagen, Crawley, U.K., http://www.qiagen.com). Total RNA was reverse transcribed using specific miRNA primers provided with TaqMan miRNA assays or reverse transcribed using random primers for mRNA expression analysis. Human miRNAs were analyzed using TaqMan miRNA assays (Applied Biosystems). FirstChoice Human Total RNA Survey Panel (Applied BioSystems, Foster City, CA, http://www.appliedbiosystems.com) was analyzed to determine miRNA expression.

### Northern Blotting

Total RNA (10 μg) was resolved in a denatured 15% polyacrylamide gel and then electrotransferred to a Hybond nylon membrane (GE Healthcare, Buckinghamshire, U.K., http://gehealthcare.com). Following carbodimide-mediated cross-linking, membranes were hybridized with digoxigenin (DIG)-labeled locked nucleic acid (LNA) probes (Exiqon, Woburn, MA) at 60°C overnight. After posthybridization washes, membranes were incubated with anti-DIG-alkaline phosphatase (AP) antibody (1:10,000; Roche, Welwyn Garden City, U.K., http://www.roche-applied-science. com) in 1% blocking reagents (Roche), and signal was visualized using CDP-Star (Sigma-Aldrich) according to the manufacturer's instructions.

### Western Blotting

Total protein (20 mg) from each sample was loaded onto a precast 4%–20% gradient Tris-glycine gel (Novex; Invitrogen), resolved at 100 V for 1 hour, and then electrotransferred onto polyvinylidene difluoride membranes. Blots were blocked for 1 hour at 25°C in Tris-buffered saline plus Tween 20 plus 3% milk. Blots were incubated with mouse monoclonal anti-human Dicer (1:1000; Abcam, Cambridge, U.K. http://www.abcam.com) or mouse monoclonal anti-human glyceraldehyde-3-phosphate dehydrogenase (GAPDH) (1:1,000, Sigma-Aldrich) followed by goat polyclonal antimouse IgG (1:10,000; Sigma-Aldrich) conjugated to horseradish peroxidase. Blots were visualized using the enhanced chemiluminescence method (ECL Kit; Amersham Biosciences, Little Chalfont, U.K., http://www. amersham.com) following the manufacturer's instructions. Membranes were reprobed for GAPDH to correct for protein loading.

### Fluorescence-Activated Cell Sorting

Cells were incubated with mouse IgG1 monoclonal anti-human CD31-fluorescein isothiocyanate (FITC) (BD Pharmingen, Oxford, U.K., http://www.bdbiosciences.com) and mouse IgG2b monoclonal anti-human VE Cadherin-phycoerythrin (PE) (R&D Systems, Abingdon, U.K., http://www.rndsystems.com) or species-matched isotype control (BD Pharmingen). Cellular fluorescence was detected in a BD Caliber Flow Cytometer.

### Assessment of Nitric Oxide Production

Quantification of nitric oxide (NO) production was conducted as according to the manufacturer's instructions (Enzo Life Sciences (U.K) Ltd., Exeter, U.K., http://www.enzolifesciences.com).

### Cell Transplantation Study

The experiments involving mice were performed in accordance with the *Guide for the Care and Use of Laboratory Animals* prepared by the Institute of Laboratory Animal Resources and covered by U.K. Home Office licenses. Unilateral hind limb ischemia was induced in immunocompromised CD1-*Foxn1*^nu^ mice (Charles River, U.K.), as described [11, 39]. hESC-ECs differentiated for 14 days (10^6^ cells in 15 μl of culture medium) infected with control LV or pLNT/SFFV-premiR-99b, -181a, or -181b (*n* = 10 mice/group) were injected immediately after induction of ischemia in three equidistant sites of the ischemic adductor muscle along the projection of the femoral artery. Foot blood flow was measured at basal, 7, 14, and 21 days after ischemia by using a high-resolution laser Doppler imager system (MoorLDI2, Moor Instruments, Axminster, U.K., http://www.moor.co.uk). At 21 days postsurgery, the limbs of terminally anesthetized mice were perfusion-fixed and ischemic adductor muscles harvested. Capillary density was measured in transverse muscular section following staining with biotinylated lectin (isolectin; 1:100, Invitrogen) to recognize ECs [40, 41].

### Statistical Analysis

Prior to any statistical analysis, data were tested for and shown to exhibit Gaussian distribution. Gaussian distribution was determined by applying the Shapiro–Wilk normality test to the data. Where appropriate, values were presented as means ± SEM.

## RESULTS

### Knockdown of Dicer Reduces hESC Differentiation to Endothelial Lineage

**Figure 1:**
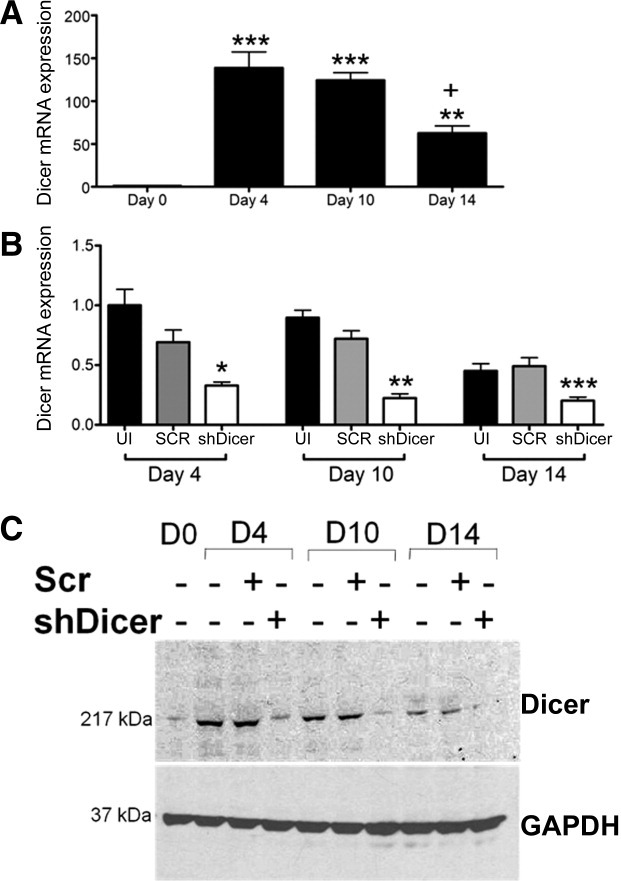
Lentiviral (LV)-mediated knockdown of Dicer in human embryonic stem-endothelial cells (hES-ECs). (A): Dicer mRNA expression during SA461 hES-EC differentiation. *Denotes significance, when compared with pluripotent D0 sample. ^+^Denotes significance, when compared with D4 and D10 hES-EC sample. (B): Dicer mRNA expression after LV-mediated knockdown. Pluripotent SA 461 hESCs (2 × 10^4^) were transduced with a multiplicity of infection of 25 prior to directed EC differentiation. Pluripotent samples (black bar), LV-shRNA SCR sequence (gray bar), and LV-shDicer sequence (white bars). Data are given as the mean ± SEM. *, *p* <.05; **, *p* <.01; ***, *p* <.001 versus the time-matched uninfected sample. (C): Western blot analysis of Dicer protein expression in SA461 hESCs. Abbreviations: GAPDH, glyceraldehyde-3-phosphate dehydrogenase; SCR, scramble; shRNA, short hairpin RNA; UI, uninfected.

We first evaluated regulation of Dicer expression during hES-EC differentiation in hESC lines SA461 and H1. Dicer was increased 130-fold after 4 days, 123-fold after 10 days, and 62-fold after 14 days of differentiation, when compared with pluripotent samples (Fig. 1A). LV-mediated knockdown of Dicer mRNA expression in hESCs was confirmed by comparison with uninfected and scrambled LV-short hairpin rna (ShRNA) controls and was sustained throughout a 14-day period. No significant difference was observed in cells infected with a LV encoding a scramble sequence, when compared with uninfected controls at any time point, whereas LV-Dicer shRNA evoked a significant suppression of Dicer mRNA at 4, 10, and 14 days postinduction of differentiation, when compared with uninfected controls (Fig. 1B). Protein expression of Dicer paralleled the mRNA expression profile. Although no notable difference was observed between uninfected and scramble sequence control samples (Fig. 1C), Dicer protein expression was reduced substantially at all times in samples subjected to LV-mediated Dicer knockdown (Fig. 1C).

**Figure 2:**
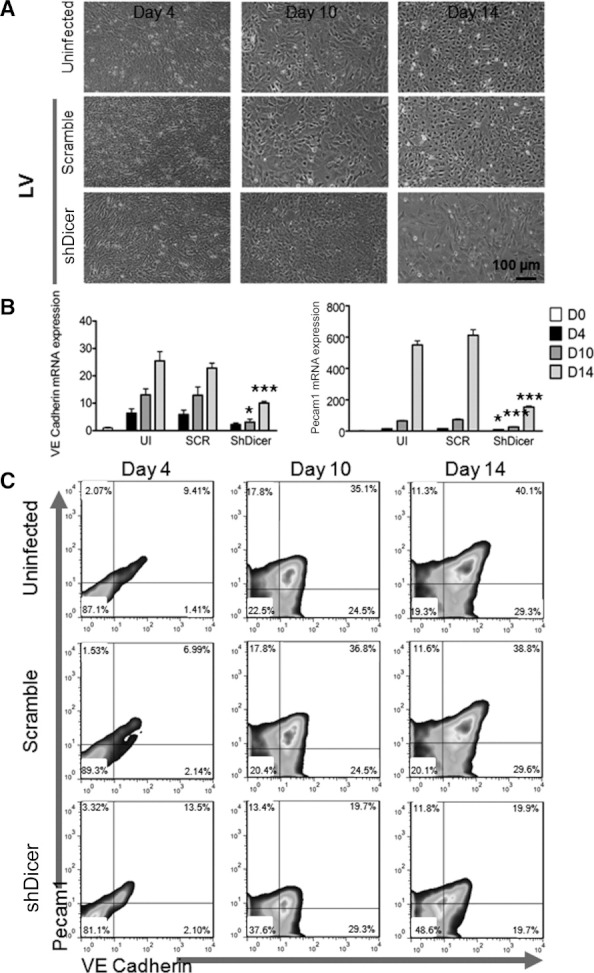
Dicer knockdown is correlated with lack of acquisition of endothelial cell (EC) phenotype. (A): Morphology of SA461 human embryonic stem cells subjected to LV-mediated Dicer knockdown or SCR sequence control (multiplicity of infection 25). Control cells (UI and SCR sequence samples) display morphological acquisition of a cobblestone-like appearance, typical of EC differentiation. Dicer knockdown prevents the appearance of morphological changes indicative of EC differentiation. Scale bar = 100 μm. (B): mRNA expression of VE Cadherin and Pecam1 in SA461 cells subjected to LV-mediated Dicer knockdown or UI and SCR sequence controls. D0 = white bars, D4 = black bars, D10 = dark gray bars, and D14 = light gray bars. Data are given as the mean ± SEM. *, *p* <.05; ***, *p* <.001 versus the time-matched uninfected sample. (C): Fluorescence-activated cell sorting analysis of increasing positive expression of VE Cadherin (FL1) and Pecam1 (FL2) observed in a time- and differentiation-dependent manner for more than 14 days of directed differentiation. LV-mediated Dicer knockdown reduced the population expressing one or both markers. Abbreviations: LV, lentiviral; SCR, scramble; UI, uninfected; VE, vascular endothelial.

**Figure 3:**
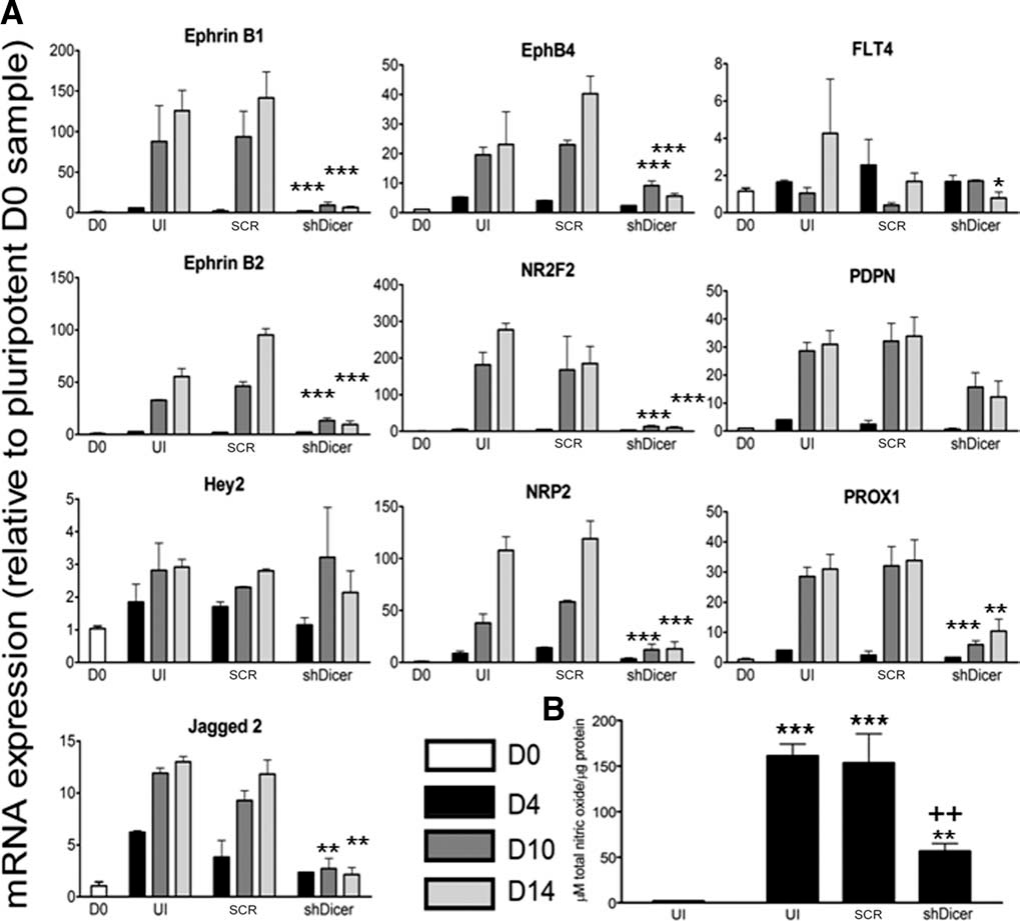
Dicer knockdown prevents the acquisition of vascular bed marker genes and reduces nitric oxide (NO) production. (A): mRNA expression of vascular bed marker genes in H1 human embryonic stem cells (hESCs). Expression of all vascular bed markers are induced with differentiation in UI and SCR sequence controls but reduced with Dicer knockdown. D0 = white bars, D4 = black bars, D10 = dark gray bars, and D14 = light gray bars. Data are given as the mean ± SEM. *, *p* <.05; **, *p* <.01; ***, *p* <.001 versus the time-matched uninfected sample. (B): NO production in D0 and D14 H1 hES-endothelial cells subjected to lentiviral-mediated Dicer knockdown or UI and SCR sequence controls. Dicer knockdown suppresses NO production. Data are given as the mean ± SEM. **, *p* <.01; ***, *p* <.001 versus the time-matched uninfected sample or ^++^, *p* <.01 versus pluripotent sample. Abbreviations: SCR, scramble; UI, uninfected.

Morphological analysis of these cells at days 4, 10, and 14 of differentiation demonstrate that reduction of Dicer was correlated with lack of morphological changes indicative of EC differentiation, namely the acquisition of a cobblestone-like morphology, as observed in all control cells (Fig. 2A). Dicer knockdown also suppressed expression of classic endothelial marker genes, VE Cadherin and Pecam1, by TaqMan (Fig. 2B) and fluorescence-activated cell sorting (FACS) analysis (Fig. 2C). Dicer knockdown abrogated mRNA expression of VE Cadherin (4.8- and 2.7-fold) and Pecam1 (7- and 3.5-fold) at days 10 and 14 postinduction of differentiation (Fig. 2B). We also used a panel of EC fate markers to assess the impact of Dicer knockdown. We observed the induction of all endothelial lineage markers in control samples but observed a propensity for expression of the arterial marker, Ephrin B1 (125-fold induction), and venous markers, NRP2 (115-fold induction) and NR2F2 (270-fold induction) at day 14 of differentiation, when compared with pluripotent samples (Fig. 3A). LV-mediated Dicer knockdown prevented the differentiation to arterial, venous, or lymphatic lineages, as determined by TaqMan analysis (Fig. 3A). Disruption of the miRNA biogenesis pathway also reduced NO production threefold in cells differentiated for 14 days, when compared with controls (Fig. 3B).

### Global miRNA Profiling of hESC-Derived ECs

**Figure 4:**
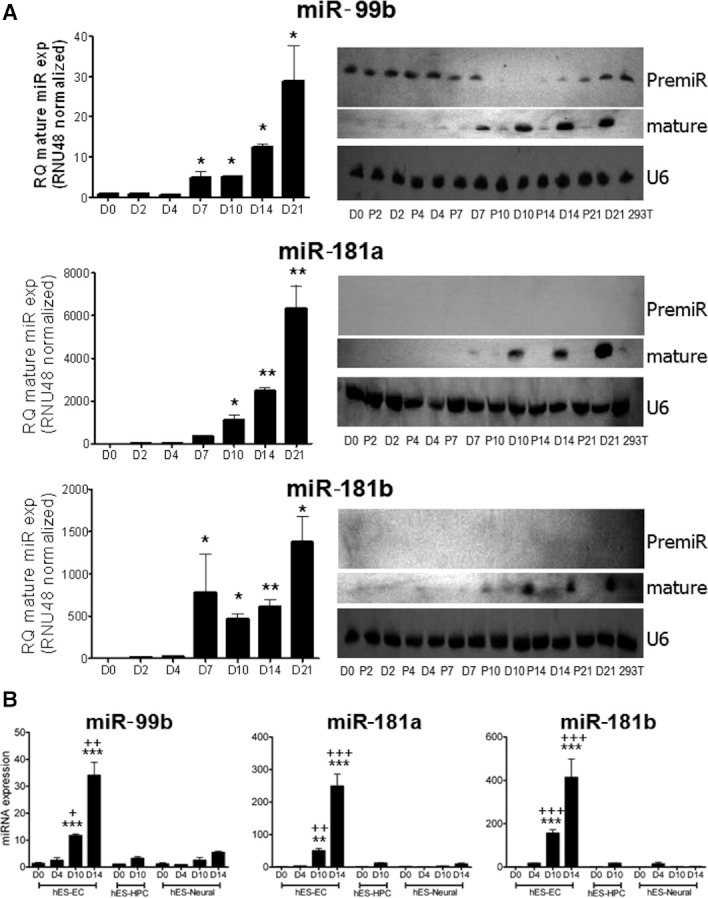
MicroRNA (miRNA) microarray validation of miRNA transcriptome at early time points of endothelial lineage specification. (A): TaqMan and Northern blot validation of microarray expression. Expression of mature miR-99b, -181a, and -181b is induced in an endothelial differentiation-specific manner, when compared with time-matched pluripotent samples in SA461 hESC line. Data are given as the mean ± SEM. *, *p* <.05; **, *p* <.01; ***, *p* <.001 versus the time-matched pluripotent sample. (B): Expression of miR-99b, -181a, and -181b in H1 pluripotent, hES-EC, hES-HPC, and hES-neural cell. Data are given as the mean ± SEM. *, *p* <.05; **, *p* <.01; ***, *p* <.001 versus pluripotent sample. ^+^Versus the time-matched differentiated sample. Abbreviations: EC, endothelial cell; hES, human embryonic stem; HPC, hematopoietic progenitor cell; RQ: relative quantification.

To assess changes in the miRNA-ome during the early stages of endothelial differentiation, the expression of 374 mature human miRNAs at days 2, 4, and 10 of endothelial differentiation (vs. pluripotent time-matched samples) in SA461 hESC line was analyzed in a two-channel microarray (Supporting Information Fig. S1A and http://www.ncbi.nlm.nih.gov/geo/query/acc.cgi?token=vtsddsqskeiwcvc&acc=GSE33675). Expression of the pluripotency-associated miRNAs miR-302a–d, -372, and -373 was significantly suppressed with progression of differentiation (Supporting Information Fig. S1B, S1C). The expression of miR-99b, -181a, and -181b was, in contrast, increased in a time- and differentiation-dependent manner compared with time-matched pluripotent samples (Fig. 4A). We validated the expression profiles in three hESC lines, SA461 (Fig. 4A), H1, and H9 cells (Supporting Information Fig. S2), directed to EC differentiation more than 21 days, when compared with time-matched pluripotent controls and observed good concordance between microarray, Q-PCR, and Northern blot analysis (Fig. 4A). We next assessed whether expression of miR-99b, -181a, and -181b would be induced in hESC-derived hematopoietic cells (mesoderm lineage) or neural cells (ectoderm lineage). hES-ECs expressed significantly elevated levels of mature miRNA than pluripotent cells or their time-matched hematopoietic or neural counterparts for all three miRNAs (Fig. 4B) confirming the selectivity to the endothelial lineage in comparison with both alternate lineages evaluated.

### Validation of EC Differentiation-Associated miRNAs in Adult ECs and Tissues

We next assessed the expression of miR-99b, -181a, and -181b in adult SVECs in comparison with SA461 hESC-ECs differentiated for 10 days and observed no differences in miR-99b expression between the aforementioned cell types (Supporting Information Fig. S3A). However, we did observe significantly less miR-181a (twofold) and -181b (threefold) expression in hESC-ECs differentiated for 10 days compared with adult ECs (Supporting Information Fig. S3A). In addition, we assessed the expression of miR-99b, -181a, and -181b across a panel of human tissues, when compared with pluripotent hESCs. We noted that miR-99b, -181a, and -181b were expressed in all tissues but that high expression was detected in brain (miR-99b, -181a, and -181b), heart (miR-99b, -181a, and -181b), and lung and thymus tissue (miR-181a and -181b) (Supporting Information Fig. S3B).

### Modulation of EC Differentiation-Associated miRNAs Potentiates EC Differentiation

**Figure 5:**
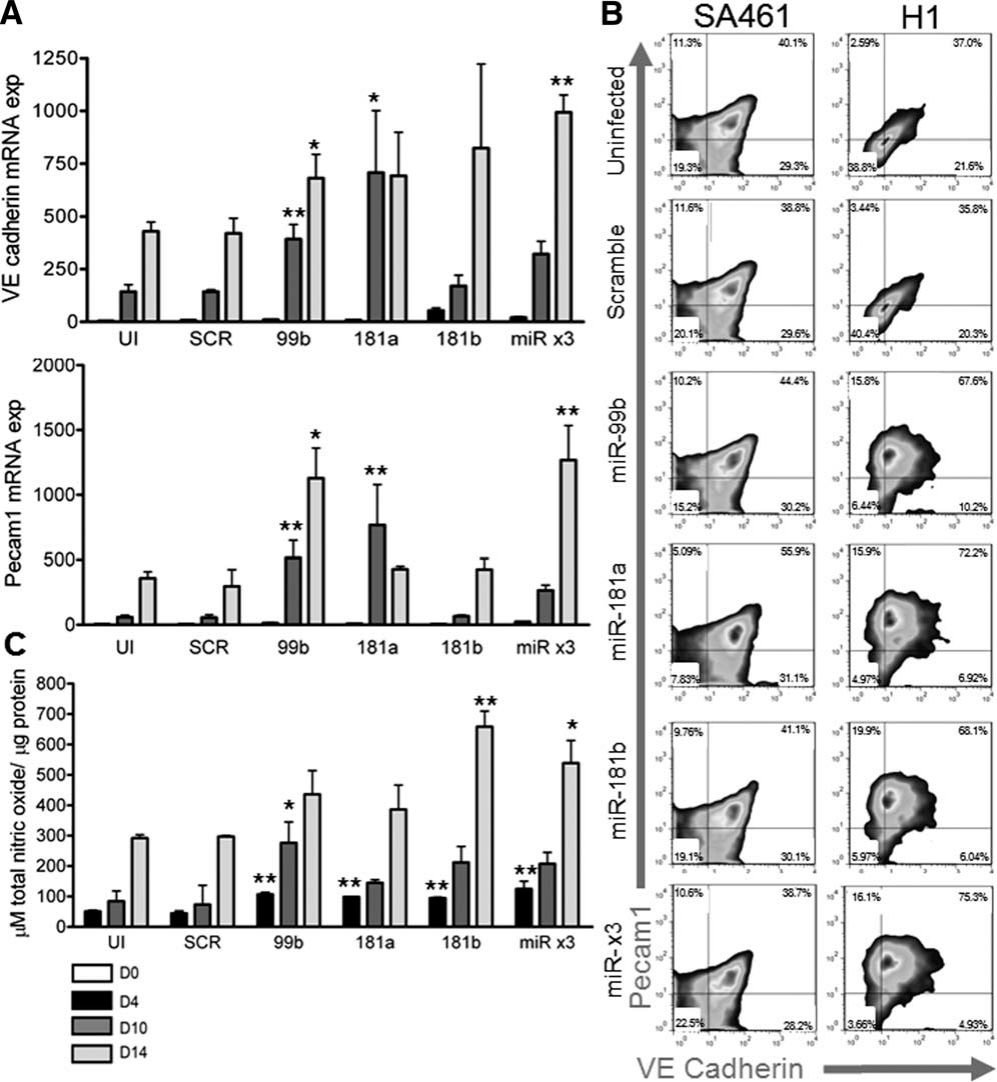
Overexpression of miRNAs potentiates endothelial cell (EC) differentiation. (A): mRNA expression of VE Cadherin and Pecam1 in H1 human embryonic stem cells (hESCs) subjected to lentiviral-mediated overexpression of miR-99b, −181a, −181b, all together (miR ×3), or UI and SCR sequence controls. (B): Fluorescence-activated cell sorting analysis of positive expression of VE Cadherin (FL1) and Pecam1 (FL2) in hES-EC subjected to 14 days of directed differentiation. (C): Nitric oxide (NO) production in hES-ECs. miR-99b, −181a, −181b, all together (miR ×3), or overexpression potentiates NO production. Data are given as the mean ± SEM. **, *p* <.01; ***, *p* <.001 versus the time-matched uninfected sample or ^++^, *p* <.01 versus pluripotent sample. D0 = white bars, D4 = black bars, D10 = dark gray bars, and D14 = light gray bars. Abbreviations: SCR, scramble; UI, uninfected; VE, vascular endothelial.

We sought to define the effect of miR-99b, -181a, and -181b modulation on EC differentiation from pluripotency. To achieve this, we used self-inactivating vesicular stomatitis virus-pseudotyped LV vectors to modulate miRNA expression by infecting pluripotent SA461 and H1 hESCs prior to directed EC differentiation. miR overexpression was achieved by inducing expression of the pre-miR sequence or scramble sequence control, using a MOI of 25. miR knockdown was achieved by inducing expression of a miRZip antisense miRNA RNA interference (RNAi) hairpin (that competitively binds the endogenous miRNA target and inhibit its function) or scramble sequence control, using a MOI of 25. Efficient modulation of each miRNA was confirmed by TaqMan in both hESC lines (Supporting Information Fig. S4). miRNA overexpression potentiated mRNA expression of EC-specific markers, Pecam1 and VE Cadherin, at days 10 and 14 of differentiation, when compared with controls (Fig. 5A). FACS analysis of Pecam1 and VE Cadherin demonstrated that H1 hESCs are more refractory to endothelial differentiation than SA461 hESCs, with 80% SA461 (mean fluorescence intensity (MFI): 14.0) and only 61% H1 (MFI: 9.0) cells expressing one or both endothelial markers at day 14 (Fig. 5B). miR overexpression elicited a greater effect in H1 cells, resulting in a 33% (miR-99b, MFI: 27.5), 34% (miR-181a, MFI: 33.5), and 33% (miR-181b, MFI: 28.9) increase in cells expressing one or both Pecam1 and VE Cadherin at day 14 of differentiation (vs. uninfected and scramble sequence control samples), when compared with a 4% (miR-99b, MFI: 15.34), 11% (miR-181a, MFI: 25.1), and 0% (miR-181b, MFI: 20.0) increase in SA461 cells (Fig. 5B). Conversely, miR inhibition did not reduce the population of cells expressing endothelial markers in either cell line (Supporting Information Fig. S5B); however, we did observe a significant reduction in Pecam1 and VE Cadherin mRNA expression, signifying discordance between transcript and protein expression profiles (Supporting Information Fig. S5B).

We also assessed whether miRNA modulation would have any effect on NO production of hESC-ECs differentiated for up to 14 days. Overexpression of miR-99b, -181a, and -181b evoked an increase in NO production in hES-ECs after 4 days of differentiation, when compared with control samples. Overexpression of miR-99b and -181b also augmented NO production at days 10 and 14, respectively (Fig. 5C). Conversely, inhibition of miR-99b, -181a, and -181b evoked a reduction in NO production, with suppression observed at day 14 (Supporting Information Fig. S5C). No additive or synergistic effect on endothelial marker expression or NO production was observed with combinatorial overexpression or knockdown of miR-99b, -181a, and -181b together (Fig. 5, Supporting Information Fig. S5).

**Figure 6:**
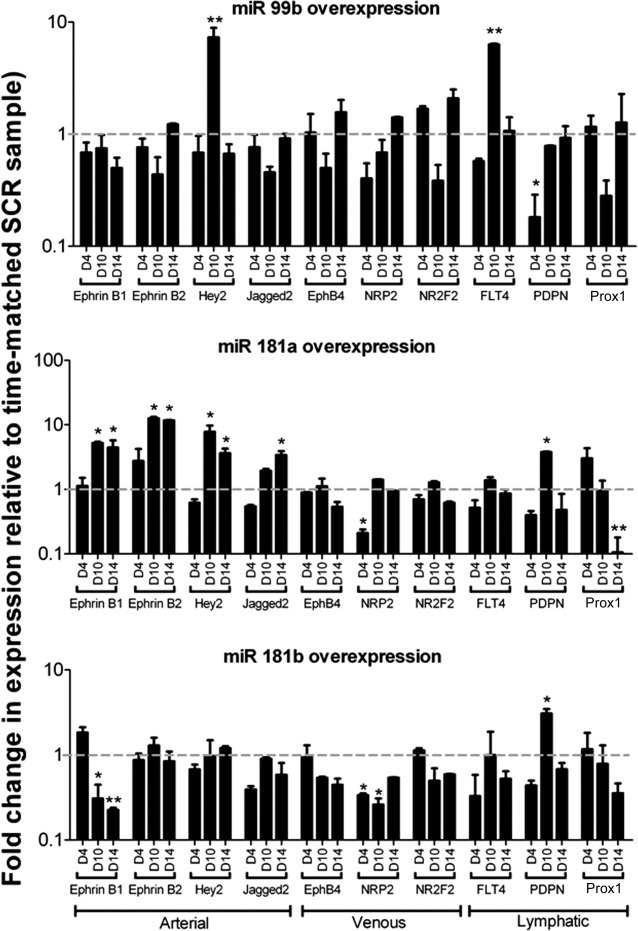
Expression of vascular bed markers after microRNA overexpression modulation. Fold expression of vascular bed marker genes in H1 human embryonic stem cells relative to time-matched lentiviral (LV)-shRNA scramble sequence controls. Data are given as the mean ± SEM. *, *p* <.05; **, *p* <.01 versus the time-matched LV-shRNA scramble sequence sample. Gray hatched line denotes scramble sequence reference value. Abbreviation: SCR, scramble; shRNA, short hairpin RNA.

We next determined whether the modulation of miRNAs miR-99b, -181a, and -181b would have an effect on the lineage specification of hESC-ECs. Overexpression of miR-99b evoked a significant induction of the arterial gene, Hey2, and the lymphatic gene, FLT4. miR-99b overexpression also suppressed expression of the lymphatic marker, podoplanin (PDPN) (Fig. 6). Inhibition of miR-99b intriguingly also augmented expression of PDPN and the arterial genes, Hey2 and Jagged2, but suppressed expression of the arterial gene, Ephrin B2, and venous genes, EphB4 and NR2F2, when compared with scrambled sequence control samples (Supporting Information Fig. S6). Overexpression of miR-181a potentiated the expression of all arterial genes analyzed, Ephrin B1, Ephrin B2, Hey2, and Jagged2 and the lymphatic gene, PDPN. miR-181a overexpression also suppressed expression of NRP2 and Prox1, when compared with control samples (Fig. 6). Inhibition of miR-181a induced expression of Prox1 and suppressed expression of Ephrin B2, when compared with control samples (Supporting Information Fig. S6). Overexpression of miR-181b potentiated the expression of the lymphatic gene, PDPN, but suppressed the expression of Ephrin B1 and NRP2, as compared to scramble sequence control samples (Fig. 6). Inhibition of miR-181b suppressed expression of the arterial genes, Ephrin B1, Ephrin B2, and Hey2 and induced expression of all lymphatic genes analyzed, FLT4, PDPN, and Prox1, when compared with control samples (Supporting Information Fig. S6). Changes in vascular bed genes were not uniform throughout the duration of the differentiation time course (Fig. 6 and Supporting Information Fig. S6).

**Figure 7:**
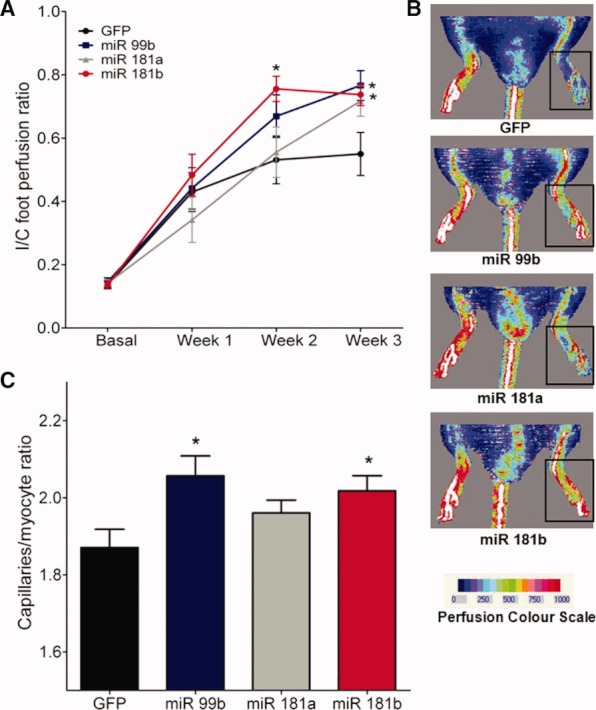
Overexpression of miRNAs improves therapeutic angiogenesis. (A): Ischemic to contralateral blood flow ratio at 7, 14, and 21 days after injection of cells in the ischemic adductor muscle. (B): Photographs show typical laser Doppler images of blood flow captured from human embryonic stem cell-endothelial cell (GFP, miR-99b, -181a, and -181b overexpression groups)-injected mice at 21 days after induction of ischemia. The squares highlight the areas of interest (feet), where blood flow was calculated to determine the ischemic/contralateral ratio. Color scale from blue to red indicates progressive increases in blood flow. (C): Capillary density of ischemic adductor muscle (21 days postischemia). Capillary density is expressed as the number of capillaries to myofiber ratio. Data are mean ± SEM; *, *p* <.05 versus control GFP expressing virus. Abbreviation: GFP, green fluorescent protein.

To determine the therapeutic potential of miR-modified hESC-ECs for vascular regeneration in vivo, day 14-differentiated H1 hESC-ECs infected with pLNT/SFFV-green fluorescent protein or pLNT/SFFV-premiR-99b, -181a, or -181b were intramuscularly injected into the ischemic adductor muscle of immunocompromised mice and hind limb blood flow recovery monitored for more than 21 days. Figure 7B shows representative Doppler images at day 21 postischemia. At 21 days postinduction of ischemia, blood flow to the ischemic foot was improved by hESC-ECs overexpressing either miR-99b or -181a, when compared with the virus control. A trend toward improved blood flow was observed with hESC-ECs overexpressing miR-181a; however, this did not attain statistical significance compared with virus control (Fig. 7A). In agreement with improved blood perfusion data, transplantation of hESC-EC subjected to miR-99b or -181b overexpression also enhanced capillary density of the ischemic adductor at 21 days postischemia, whereas miR-181a had no effect (Fig. 7C).

## DISCUSSION

This study describes the identification of three miRNAs that are expressed upon differentiation of ECs from pluripotent stem cells. We report that miRNA processing by Dicer is necessary for endothelial differentiation, evidenced by Dicer knockdown substantially reducing hESC differentiation to EC. We analyzed early stages of EC lineage commitment and the miRNA transcriptome to identify miR-99b, -181a, and -181b specifically upregulated during differentiation. Moreover, we report that augmentation of miRNAs by gene transfer can potentiate the endothelial differentiation capacity in a hESC line that evokes relatively poor differentiation to ECs. Finally, we demonstrate that the transplantation of hESC-ECs overexpressing either miR-99b or -181b into the muscle of immunodeficient mice subjected to hind limb ischemia promoted therapeutic neovascularization and blood flow recovery.

With respect to in vitro differentiation from pluripotent stem cells, the role of miRNAs has been implicated in studies using Dicer- or Drosha-deficient mouse ESCs. Dicer- or Drosha-deficient ESCs were unable to generate mature miRNAs, which subsequently impeded differentiation [23, 42, 43]; however, this has as yet not been reported in hESCs. We report herein the first demonstration that LV-mediated knockdown of Dicer abrogated the potential of pluripotent hESCs to differentiate to an EC lineage (Fig. 2) highlighting that the canonical biogenesis of mature miRNAs is likely required for successful endothelial differentiation. Herein, we also report that Dicer knockdown suppresses the production of NO in hESC directed to EC lineage (Fig. 3). This is in agreement with our previous data demonstrating a differentiation-specific increase in EC marker gene expression concordant with a functional EC phenotype in vitro and in vivo and the ability to produce and respond to an NO stimulator [11]. Recent additional studies have reported that abrogation of Dicer is capable of preventing ES differentiation toward other lineages [44–48]. We have focused on hES-EC differentiation, but it is likely that Dicer suppression would impair human pluripotent cell differentiation to other lineages similar to murine ESCs.

We reasoned that lineage-specific miRNAs must be processed and contribute to the master regulation of the earliest stages of lineage specification and commitment. We have previously demonstrated that the expression of angiogenesis-associated mature miRNAs (miR-126, -130a, -133a, -133b, and -210) was present at 10-day postdifferentiated functional hES-ECs [11]. With this in mind, we sought to identify miRNAs expressed at earlier stages of lineage commitment to EC. We differentiated hESCs toward an EC lineage using our previously reported serum- and feeder-free protocol [11]. This protocol circumvented the need for an embryoid body formation with cells differentiating in a uniform manner, thereby providing a unique opportunity to analyze the miRNA component at early stage of lineage commitment without the need to deplete cells from the other germ lineages. In agreement with previous studies, we identified the expression of the pluripotency-associated miRNA clusters 302 and 372/373 as present in our pluripotent hESC samples [12, 13]. We also validated the substantial loss of differentiation-associated miR-302a–d, -372, and -373 in agreement with previous studies [12, 13]. We identified and validated the expression of miR-99b, -181a, and -181b in the early stages of EC differentiation from three pluripotent hESC lines. miR-99b is an intergenic miRNA and transcribed in a cluster with miR-125a and Let7e. This is in agreement with our previous finding that members of the Let7 family were induced upon directed differentiation to ECs [11]. Furthermore, we also identified miR-125a-5p as being highly expressed in the early stage of EC lineage specification (http://www.ncbi.nlm.nih.gov/geo/query/acc.cgi?token=vtsdd sqskeiwcvc&acc=GSE33675). miR-181a and -181b are both predicted to be expressed from two genomic hairpin loci, miR-181a/b-1 and -181a/b-2 (www.mirbase.org). The mature sequences are identical regardless of cleavage by Dicer from pre-miR-181a/b-1 or -2. miR-181a and -181b are both intragenic, but the genes that they reside in currently have no known function. We subsequently confirmed the expression of miR-99b, -181a, and -181b in adult human SVECs, with no notable differences observed in mature miR-99b expression levels between the adult ECs and SA461 hES-ECs differentiated for 10 days (Supporting Information Fig. S3A). We did observe significantly less miR-181a and -181b between day 10-differentiated hES-ECs and adult SVECs although levels at day 10 were easily detected by Northern blot analysis (Supporting Information Fig. S3A). The identification of miR-99b [49], -181a [50], and -181b [19] is consistent with previous miR profiling data performed in ECs, but to our knowledge none have been associated with differentiation from pluripotency to endothelial lineage. miR-181b has also previously been identified as expressed in CD34+ cells during hematopoietic differentiation from pluripotent cells [51]. CD34 is expressed on mesoderm and early ECs [52] providing further evidence for a miR-181b role in early-stage commitment to EC differentiation. In addition, we have demonstrated that there is very low expression of miR-99b, -181a, and -181b in cells that are differentiated toward neural (ectopic) or hematopoietic (mesoderm) lineages; however, this is greatly potentiated in cells directed toward EC lineage (Fig. 4). To this end, it remains likely that miR-99b, -181a, and -181b are also expressed to a certain extent on cells of mesoderm origin, for example, hematopoietic cells owing to the common precursor that HSC and EC develop through [53]. However, this does highlight that in some capacity miR-99b, -181a, and -181b could be acting to promote cell cycle and generic differentiation progression from the pluripotent state, as previously reported for miR-195 and -372 [54].

We sought to determine whether modulation of miR-99b, -181a, or -181b would elicit any effect of the differentiation capacity of pluripotent stem cells toward an EC lineage. Overexpression of the premiR sequence for miR-99b, -181a, and -181b induced a significant increase in mature miR-99b, -181a, and -181b expression, respectively, thus signifying efficient processing of pre-miRNAs (Supporting Information Fig. S4). miR-99b and -181a augmentation was capable of significantly increasing the vascular EC marker genes, Pecam1 and VE Cadherin, at the transcript level in both hESC lines (Fig. 6). No additional benefit was observed by overexpressing all three miRNAs in combination. Furthermore, we report that upon analysis of the cell population expressing both proteins and ability to produce NO, we noted that there was an increased response to miRNA overexpression for all three miRNAs, including miR-181b that did not evoke a significant response at the mRNA level, in the H1 hESC line, when compared with SA461 cells (Fig. 5). This was encouraging as we had also observed a substantial difference in the baseline efficiency of the hESC lines to acquire an endothelial phenotype (expression of one or both markers), with H1 cells proving more refractory than SA461 cells when exposed to our EC differentiation protocol (Fig. 5), in agreement with a previous study examining the differentiation heterogeneity between lines [55]. We acknowledge that expression of one marker alone is not conclusive demonstration of EC lineage, but it may indicate a proportion of cells that are still differentiating to a terminally differentiated, bona fide EC. Indeed, in our previous manuscript [11], we reported that the percentage of SA461 cells expressing both markers increased from days 14 to 21 of differentiation, this is in keeping with the data produced herein. Furthermore, it remains possible that we are unable to improve the differentiation efficiency of SA461 hES owing to the high base level EC differentiation efficiency (as determined by cells expressing one or more EC markers and MFI values), whereas with H1 cells there remains potential to enhance the differentiation, in this instance by modulating miRNA expression. It is therefore feasible that a finite EC differentiation capacity exists regardless of the hES background. Therefore, we suggest that pluripotent stem cell lines that are more refractory to differentiation protocols may benefit from the modulation of miRNAs to enhance the differentiation efficiency, but this requires analysis in other lines for confirmation. In addition, we observed significantly improved therapeutic angiogenesis in a mouse model of peripheral ischemia after transplantation of hESC-ECs differentiated for 14 days, which overexpressed either miR-99b or -181b but interestingly not miR-181a (Fig. 7). This miRNA-mediated increase in neoangiogenesis efficiency may be a combination of a direct cell effect of transplanted cells and miR-mediated proangiogenic paracrine mechanisms. These results show that overexpression of each of the individual miRNAs is capable of enhancing the vascular endothelial phenotype but not all will evoke a significant response at transcript, protein, and functional level, indicating that their modus operandi in EC biology is considerably complex. Knockdown studies also failed to elucidate the role of miR-99b, -181a, and -181b, with a significant reduction observed in vascular endothelial markers at the transcript level and a reduction in NO production, but no effect on the cell population expressing endothelial marker proteins (Supporting Information Fig. S5).

Previous studies have not provided evidence for a single miRNA possessing a pivotal role in the governance of EC fate decisions despite the demonstration of differentially expressed miRNAs during endothelial differentiation in vitro [24, 25]. We postulate that cell specification during the later stages of development may be controlled by miRNAs. For instance, miR-126 is not required for endothelial differentiation [27] but has been reported to inhibit erythropoiesis in CD34+ cells retaining the potential to develop toward either EC lineage or hematopoietic cell lineage [51], indicating miRNAs may negatively regulate further lineage specification. This has also recently been demonstrated in ECs, with miR-181a reported to directly target Prox1, the key regulator of lymphatic EC identity, [56, 57] and miR-181a overexpression directing lymphatic ECs toward a blood vascular phenotype [50].

This is in agreement with the data presented herein, with miR-181a knockdown inducing expression of Prox1 and overexpression augmenting the expression of the arterial-specific genes, EphrinB1, EphrinB2, Jagged2, and Hey2 [58], and inhibiting the expression of Prox1 [56] (Fig. 6). This increase in arterial gene expression is perhaps surprising considering that we observed no significant improvement in therapeutic angiogenesis with miR-181a overexpressing hES-ECs. Overexpression and knockdown strategies for miR-99b and -181b failed to determine a similar pattern; however, speculatively, it is possible that these miRs may be more involved in the function of hES-ECs. We observe improvement in hES-EC NO production at later differentiation time points for overexpression of miR-99b and -181b than for -181a (Fig. 5). This may in turn elicit a more potent response in vivo, potentially accounting for the increased improvement in neoangeogenesis. Equally, it remains possible that the expression of these miRNAs is increased as a consequence of endothelial differentiation, as opposed to their expression governing differentiation, and that their role in cell specification remains to be fully elucidated. To understand the functional role of these or any subsequently identified miRNAs in endothelial fate determination, it will be necessary to identify and validate target genes involved in developmental specification. There are several predicted targets for miRNAs miR-99b, -181a, and -181b; however, most of these targets have not been experimentally validated. Our study is impaired toward identifying targets of miR-99b, -181a, and -181b as most of these predicted targets will not be expressed in pluripotent, mesoderm, or ECs and their precursors. It is likely that the increased expression of these and other miRNAs serves to inhibit the induction of genes, which may facilitate development or differentiation to alternative germ layers or mesoderm cell lineages.

## CONCLUSIONS

In conclusion, this is the first study to demonstrate that miR-99b, -181a, and -181b are upregulated during hESC differentiation to ECs and can potentiate EC differentiation from pluripotent hESCs; miR-99b and -181a/b overexpression can improve the differentiation efficiency of hESC lines, which are refractory to specified lineage differentiation and enhance postischemic neoangiogenesis induced by hESC-ECs. Furthermore, elucidation of the role of miRNAs and the use of miRNA signature panels in hES-EC differentiation will aid the development of refining new approaches for cardiovascular disease profiling and cell therapy.

## Notes

Supplementary Figure 1: Schematic of miRNA microarray and validation of pluripotency-associated miRNAs. A: Schematic of design of the miRNA microarray study investigating miRNAs in hES cells subject to endothelial differentiation or pluripotent time-matched controls. Each circle represents cell groups (n = 6/group), with endothelial differentiation in green and pluripotent time matched samples in red. Each arrow represents a two-channel microarray experiment and heat map showing the expression level of all miRNA in the global profiling protocol. B: TaqMan validation of microarray expression. Expression of mature miR-302a-d, miR-372 and miR-373 is suppressed in an endothelial differentiation-specific manner, as compared to time-matched SA461 pluripotent samples. Data are given as the mean±SEM. * P<0.05, **P<0.01, and ***P<0.001 vs the time-matched pluripotent sample. C: Northern blot validation of microarray expression for miR-302c in SA461 hES cells.

Supplementary Figure 3: A: Expression of miR-99b, miR-181a and miR-181b in SA461 pluripotent D0 (white bars), D10 hES-EC (black bars), and adult SVEC cells (dark grey bars). Data are given as the mean±SEM. * P<0.05, **P<0.01, and ***P<0.001 vs Day 10 hES-EC sample. B: Expression of miR-99b, miR-181a and miR-181b across a human tissue panel, compared to SA461 pluripotent D0.

Supplementary Figure 4: Lentiviral-mediated transfer of premiR or miR Zip expression induces efficient modulation of mature miRNAs. Expression of mature miRNAs after LV-mediated expression of premiR sequences, or a miRZipTM anti-sense microRNA RNAi hairpin to miR-99b, miR-181a or miR- 181b. 2 × 104 SA461 pluripotent hES cells were transduced with MOI of 25 prior to directed EC differentiation. Expression is induced across time in a differentiationspecific manner. Expression is significantly augmented in response to LV-mediated overexpression of miR-99b, miR-181a and miR-181b, and suppressed in response to LV-mediated overexpression of a miRZipTM anti-sense microRNA RNAi hairpin, miR- 99b, miR-181a or miR-181b as compared to uninfected and scramble sequence controls (MOI 25). Data are given as the mean±SEM. * P<0.05, **P<0.01, and ***P<0.001 vs the time-matched uninfected sample.

Supplementary Figure 5: Knockdown of miRNAs has limited effect on EC differentiation. A: mRNA expression of VE Cadherin and Pecam1 in H1 hES cells subject to LVmediated suppression of miR-99b, miR-181a, miR-181b, all together (miR ×3), or uninfected and scramble sequence controls. B: FACS analysis of positive expression of VE Cadherin (FL1) and Pecam1 (FL2) in hES-EC subject to 14 days of directed differentiation. C: NO production in hES-EC cells. miR-99b, miR-181a, miR-181b or all together (miR ×3) overexpression suppresses NO production. Data are given as the mean±SEM. **P<0.01, and ***P<0.001 vs the time-matched uninfected sample or ++P<0.01 vs pluripotent sample. D0 = white bars D4 = black bars, D10 = dark grey bars, D14 = light grey bars.
