# Supplementary figures and images for: Role of MicroRNAs 99b, 181a, and 181b in the Differentiation of Human Embryonic Stem Cells to Vascular Endothelial Cells
Source: Stem Cells. 2012 Jan 9;30(4):643–54. doi: 10.1002/stem.1026 (PMC3490385; doi:10.1002/stem.1026)

# H1

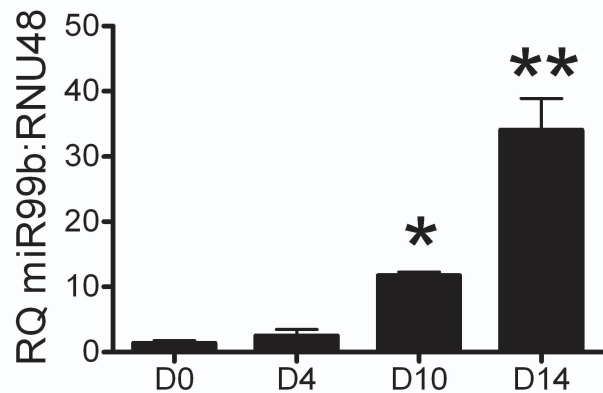

# H9

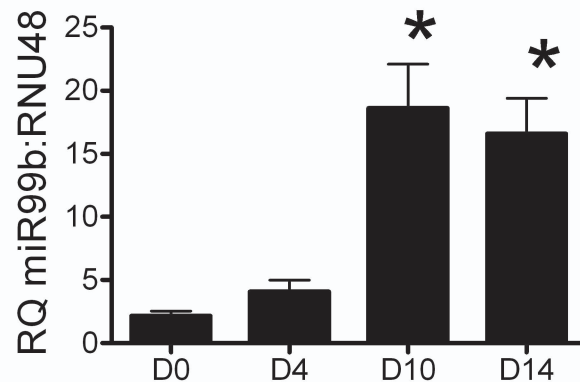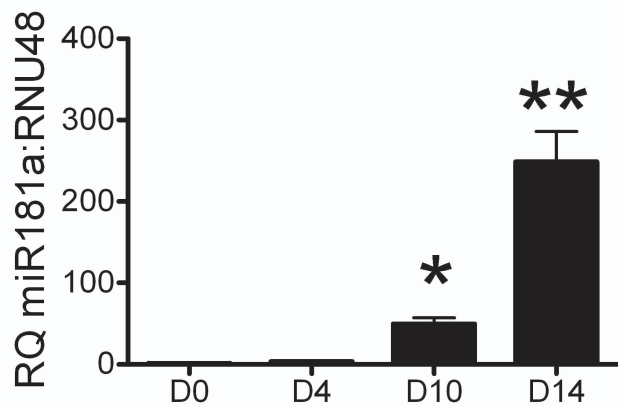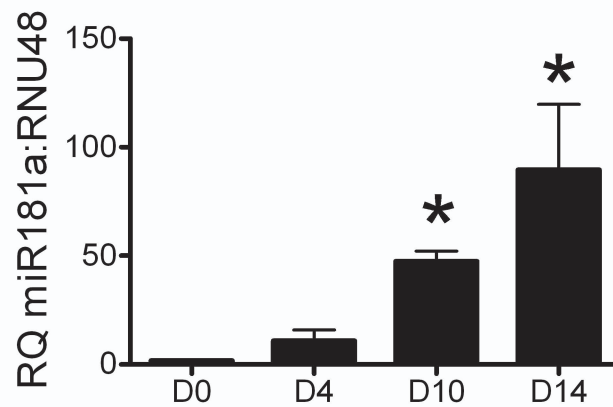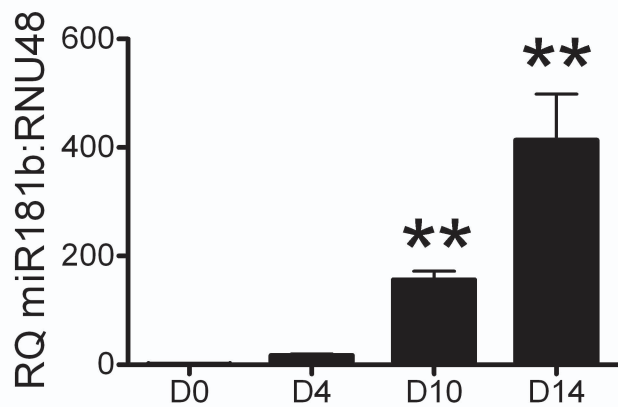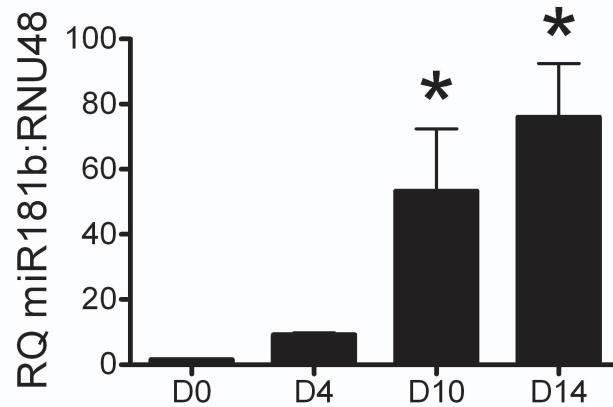

Supplement: Supplementary file 1 — Supplementary Figure 1: Schematic of miRNA microarray and validation of pluripotency-associated miRNAs. A: Schematic of design of the miRNA microarray study investigating miRNAs in hES cells subject to endothelial differentiation or pluripotent time-matched controls. Each circle represents cell groups (n = 6/group), with endothelial differentiation in green and pluripotent time matched samples in red. Each arrow represents a two-channel microarray experiment and heat map showing the expression level of all miRNA in the global profiling protocol. B: TaqMan validation of microarray expression. Expression of mature miR-302a-d, miR-372 and miR-373 is suppressed in an endothelial differentiation-specific manner, as compared to time-matched SA461 pluripotent samples. Data are given as the mean±SEM. * P<0.05, **P<0.01, and ***P<0.001 vs the time-matched pluripotent sample. C: Northern blot validation of microarray expression for miR-302c in SA461 hES cells. [file stem0030-0643-SD1.pdf]

SA461

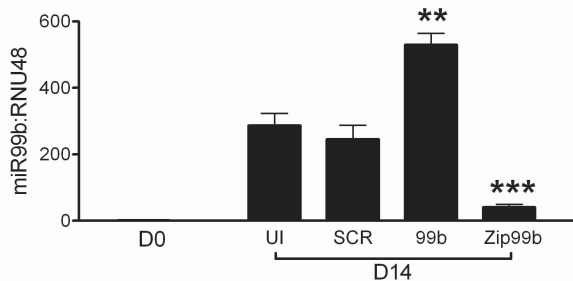

H1

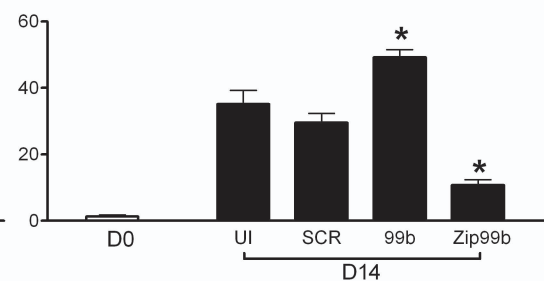

miR modulation

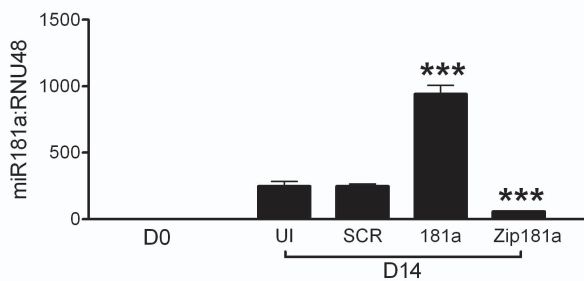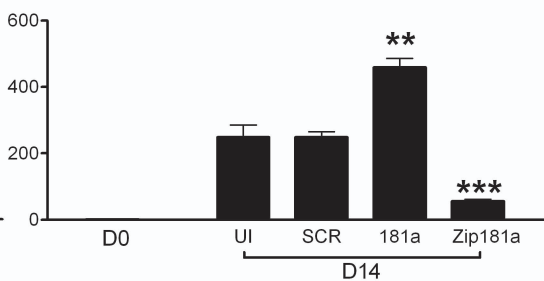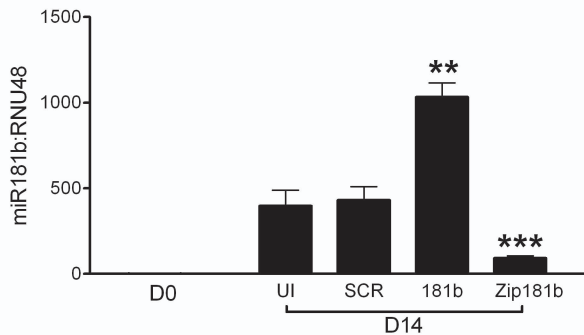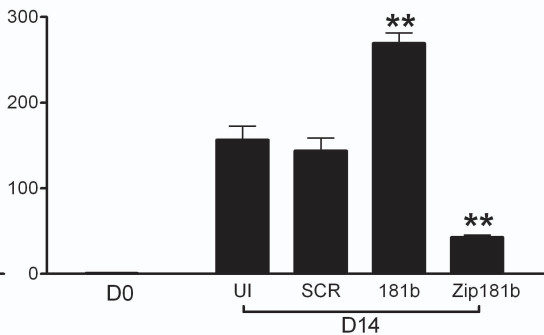

Supplement: Supplementary file 2 — Supplementary Figure 3: A: Expression of miR-99b, miR-181a and miR-181b in SA461 pluripotent D0 (white bars), D10 hES-EC (black bars), and adult SVEC cells (dark grey bars). Data are given as the mean±SEM. * P<0.05, **P<0.01, and ***P<0.001 vs Day 10 hES-EC sample. B: Expression of miR-99b, miR-181a and miR-181b across a human tissue panel, compared to SA461 pluripotent D0. [file stem0030-0643-SD2.pdf]

Fold change in expression relative to time-matched SCR sample)

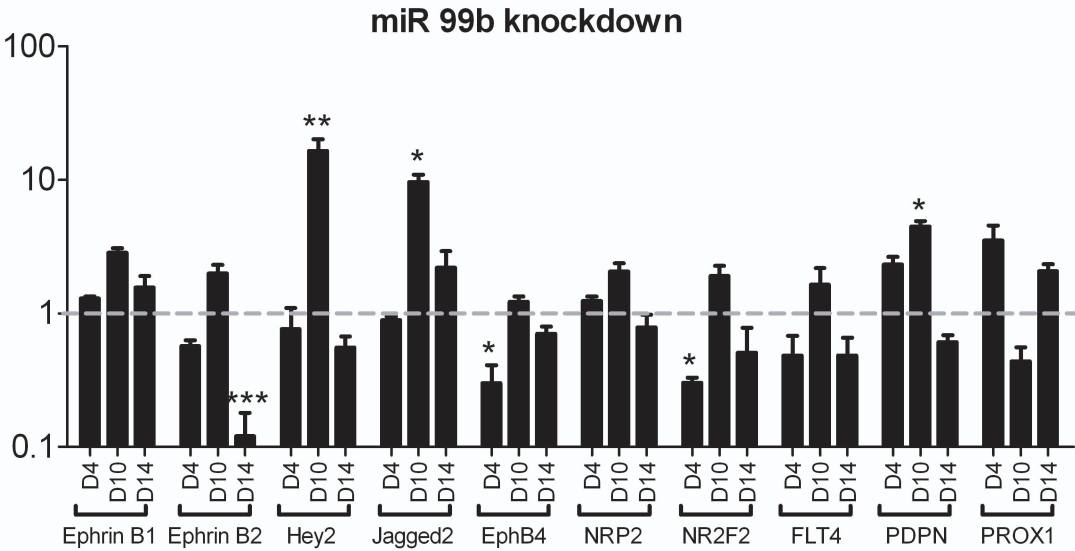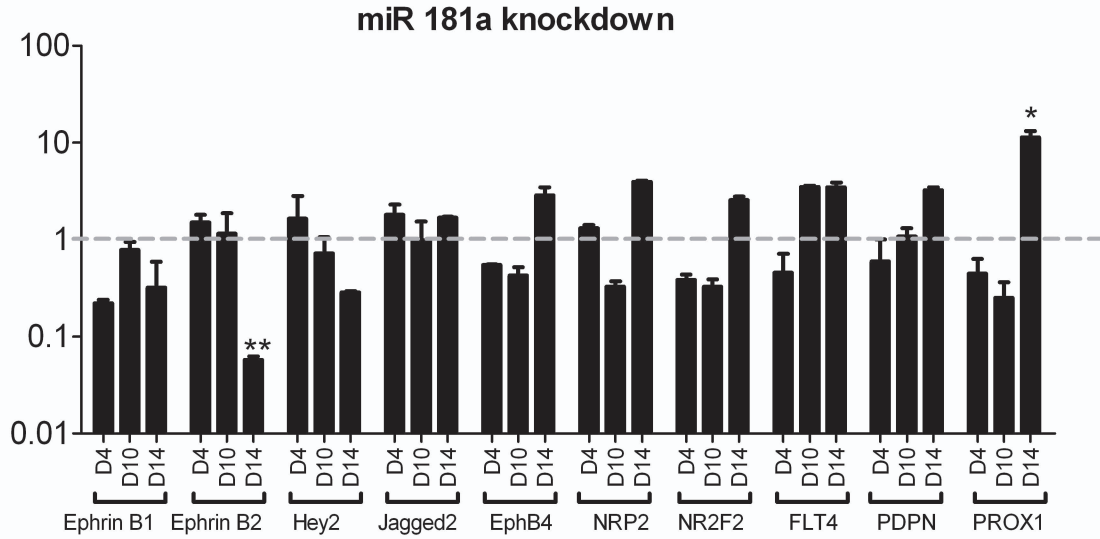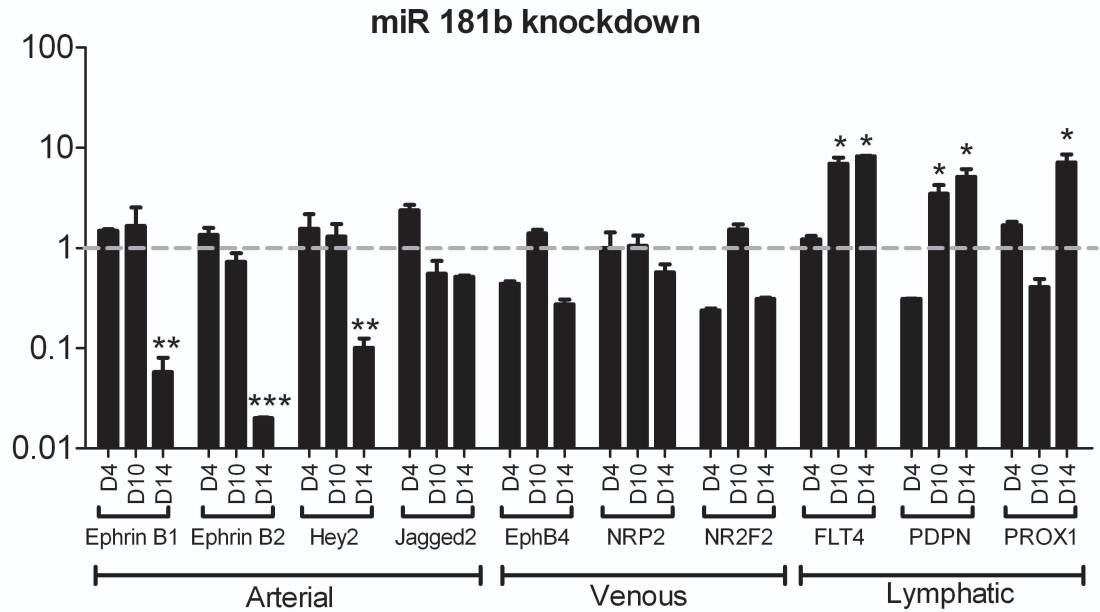

Supplement: Supplementary file 3 — Supplementary Figure 4: Lentiviral-mediated transfer of premiR or miR Zip expression induces efficient modulation of mature miRNAs. Expression of mature miRNAs after LV-mediated expression of premiR sequences, or a miRZipTM anti-sense microRNA RNAi hairpin to miR-99b, miR-181a or miR- 181b. 2 × 10^4^ SA461 pluripotent hES cells were transduced with MOI of 25 prior to directed EC differentiation. Expression is induced across time in a differentiationspecific manner. Expression is significantly augmented in response to LV-mediated overexpression of miR-99b, miR-181a and miR-181b, and suppressed in response to LV-mediated overexpression of a miRZipTM anti-sense microRNA RNAi hairpin, miR- 99b, miR-181a or miR-181b as compared to uninfected and scramble sequence controls (MOI 25). Data are given as the mean±SEM. * P<0.05, **P<0.01, and ***P<0.001 vs the time-matched uninfected sample. [file stem0030-0643-SD3.pdf]

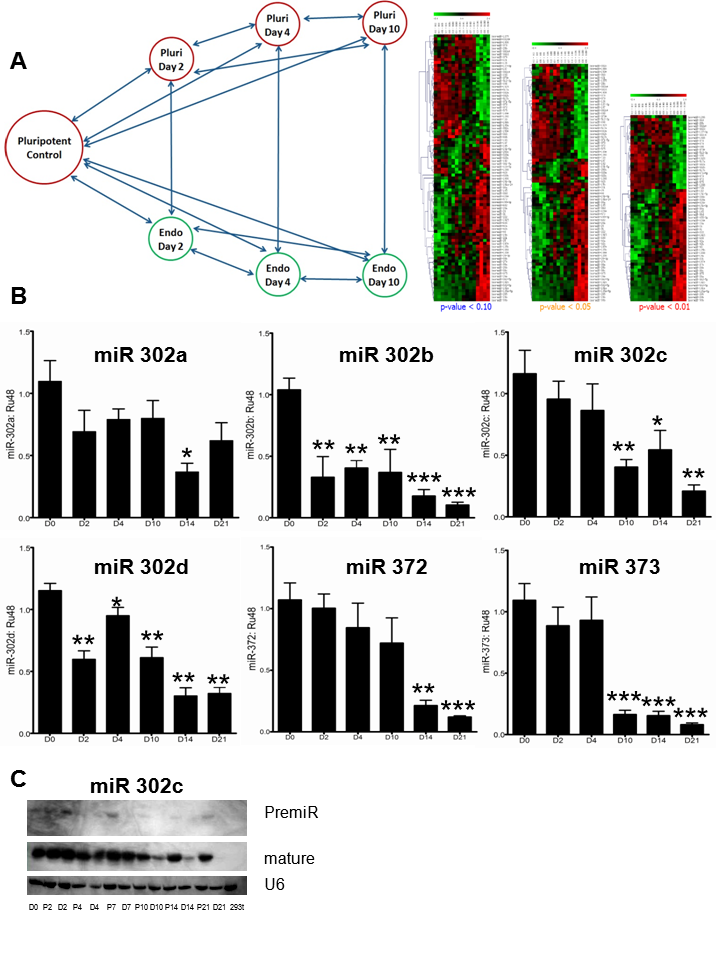

Supplement: Supplementary file 5 [file stem0030-0643-SD1.tif]

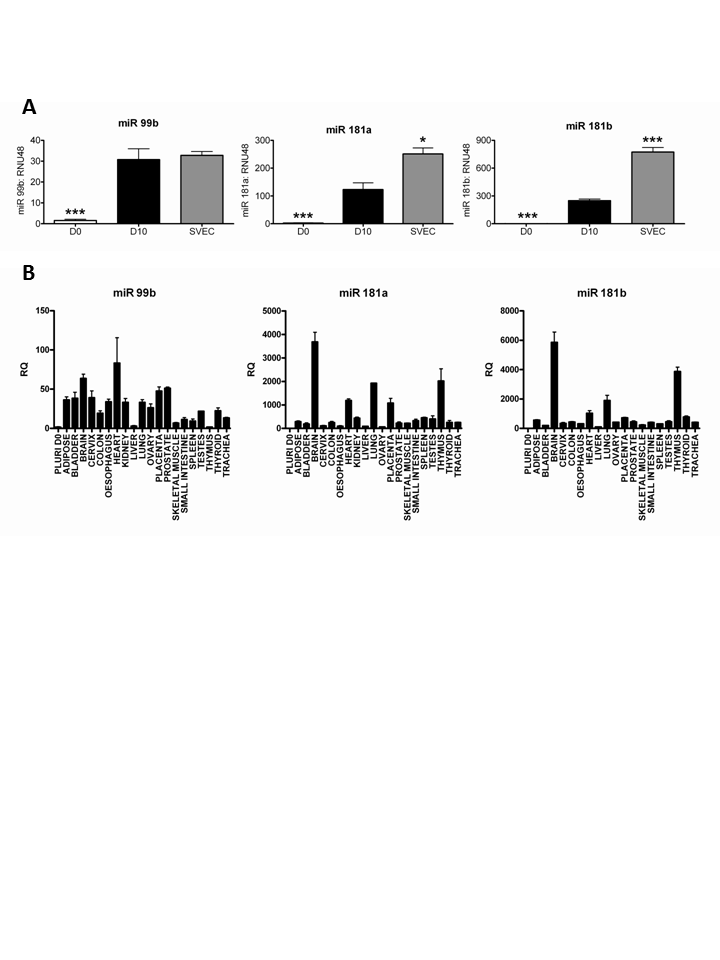

Supplement: Supplementary file 6 [file stem0030-0643-SD2.tif]

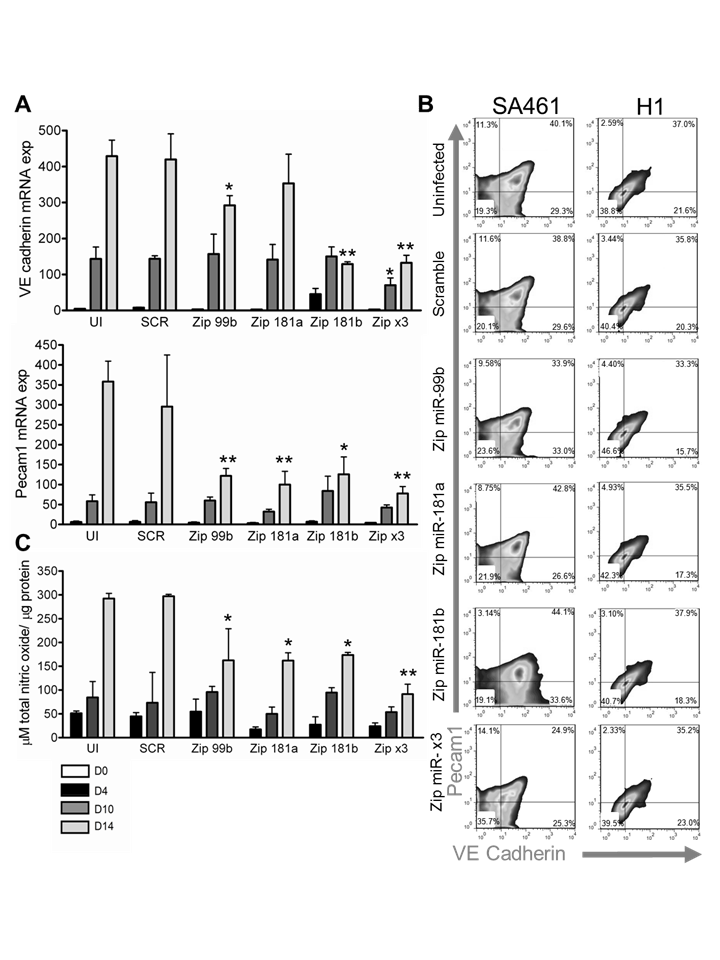

Supplement: Supplementary file 7 [file stem0030-0643-SD3.tif]
