# Supplementary material for: Role of MicroRNAs 99b, 181a, and 181b in the Differentiation of Human Embryonic Stem Cells to Vascular Endothelial Cells
Source: Stem Cells. 2012 Jan 9;30(4):643–54. doi: 10.1002/stem.1026 (PMC3490385; doi:10.1002/stem.1026)
Supplement: Supplementary file 4 — Supplementary Figure 5: Knockdown of miRNAs has limited effect on EC differentiation. A: mRNA expression of VE Cadherin and Pecam1 in H1 hES cells subject to LVmediated suppression of miR-99b, miR-181a, miR-181b, all together (miR ×3), or uninfected and scramble sequence controls. B: FACS analysis of positive expression of VE Cadherin (FL1) and Pecam1 (FL2) in hES-EC subject to 14 days of directed differentiation. C: NO production in hES-EC cells. miR-99b, miR-181a, miR-181b or all together (miR ×3) overexpression suppresses NO production. Data are given as the mean±SEM. **P<0.01, and ***P<0.001 vs the time-matched uninfected sample or ++P<0.01 vs pluripotent sample. D0 = white bars D4 = black bars, D10 = dark grey bars, D14 = light grey bars. [file stem0030-0643-SD4.pdf]

Supplementary Table 1
PremiR sequences.

**miRNA**	**5’ – 3’**	**3’ – 5’**
**miR-99b**	AAGCTTGGATCCACCATGGGGCACCCACCCGTAGAACCG<br>ACCTTGCGGGGCCTTCGCCGCACACAAGCTCGTGTCTGT<br>GGGTCCGTGTC TAG TTTTTT CTCGAG GATATC	TTCGAACCTAGGTGGTACCCCGTGGGTGGGCATCTTGG<br>CTGGAACGCCCCGGAAGCGGCGTGTGTTCGAGCACAGA<br>CACCCAGGCACAG ATC AAAAAA GAGCTC CTATAG
**miR-181a**	AAGCTTGGATCCACCATGGAGAAGGGCTATCAGGCCAGC<br>CTTCAGAGGACTCCAAGGAACATTCAACGCTGTCGGTGAG<br>TTTGGGATTTGAAAAAACCACTGACCGTTGACTGTACCTT<br>GGGGTCCTTA TAG TTTTTT CTCGAG GATATC	TTCGAACCTAGGTGGTACCTCTTCCCGATAGTCCGGTCG<br>GAAGTCTCCTGAGGTTCCTTGTAAGTTGCGACAGCCACT<br>CAAACCCTAAACTTTTTTGGTGACTGGCAACTGACATGG<br>AACCCCAGGAAT ATC AAAAAA GAGCTC CTATAG
**miR-181b**	AAGCTTGGATCCACCATGGCCTGTGCAGAGATTATTTTTTA<br>AAAGGTCACAATCAACATTCATTGCTGTCGGTGGGTTGAA<br>CTGTGTGGACAAGCTCACTGAACAATGAATGCAACTGTGG<br>CCCCGCTT TAG TTTTTT CTCGAG GATATC	TTCGAACCTAGGTGGTACCGGACACGTCTCTAATAAAAA<br>ATTTTCCAGTGTTAGTTGTAAGTAACGACAGCCACCCAA<br>CTTGACACACCTGTTCGAGTGACTTGTTACTTACGTTGAC<br>ACCGGGGCGAA ATC AAAAAA GAGCTC CTATAG
